# Effect of probiotics on the intestinal microbiota of patients with giardiasis and ascariasis

**DOI:** 10.25122/jml-2022-0191

**Published:** 2022-10

**Authors:** Oleksandra Yaroslavivna Pryshliak, Andrii Liubomyrovych Protsyk, Mariana Volodymyrivna Semaniv, Oleksandr Petrovych Boichuk, Petro Romanovych Gerych

**Affiliations:** 1 Department of Infectious Diseases and Epidemiology, Ivano-Frankivsk National Medical University, Ivano-Frankivsk, Ukraine; 2 Department of Microbiology, Virology and Immunology, Ivano-Frankivsk National Medical University, Ivano-Frankivsk, Ukraine; 3 Department of Internal Medicine and Allergology, Ivano-Frankivsk National Medical University, Ivano-Frankivsk, Ukraine

**Keywords:** giardiasis, ascariasis, mixed invasion, dysbiosis, probiotic

## Abstract

Recently, many cases of mixed invasion by Giardia and ascarids have been registered. Gastrointestinal lesions in patients are often accompanied by dysbiotic changes. The aim was to study the effect of probiotics containing *Saccharomyces bouvardias CNCM I-745* in the complex therapy of patients with giardiasis, ascariasis, and mixed invasion. 90 patients with giardiasis, ascariasis and mixed invasion were divided into 3 groups, depending on the type of invasion. Each group was divided into two subgroups, depending on the treatment (basic treatment and treatment with probiotics). When studying the state of the intestinal microbiota, the following changes were detected in all patients before treatment. The content of *Bifidobacterium spp*., *Lactobacillus spp*., *Enterococcus spp*. and the total content of *E. coli* was reduced. At the same time, there was an increase in the content of *Peptostreptococcus spp*., *Bacteroides spp*., *E. coli* with low enzymatic properties, hemolytic *E. coli*, opportunistic *Enterobacteriaceae spp*., *Candida spp*. and *Staphylococcus spp*. Under the influence of treatment, the subgroup with probiotics addition to the basic treatment, was more effective for all types of invasions. The use of probiotics containing *Saccharomyces bouvardias CNCM I-745* in the complex therapy of patients with mixed invasion of giardiasis and ascariasis increased treatment efficiency following a significant improvement in intestinal microbiota.

## INTRODUCTION

Parasitic diseases constitute a significant proportion of the burden of infectious diseases, and the prevalence in Ukraine remains high. The most common intestinal parasitic diseases are giardiasis and ascariasis. Recently, more and more cases of mixed invasion by these pathogens have been registered [1, 2]. The clinical picture of mixed invasion by *Ascaris lumbricoides* and *Giardia lamblia* is characterized by a more severe course, and the number of complications increases, including changes in the intestinal microbiota [3, 4]. Many scientists have proven that intestinal dysbiosis is not only a concomitant clinical and laboratory syndrome of major somatic gastrointestinal tract pathology but can also manifest itself as the onset of many pathological conditions, especially in young children [5–7]. Prolonged giardiasis and ascariasis in the human body lead to disorders of the entire digestive system. Mechanical damage to the small intestine mucous membrane and destruction of the glycocalyx contributes to the inoculation of opportunistic *Enterobacter*ia and pathogenic microbiota with the development of dysbiosis [7–9]. The primary modern method of pathogenetic treatment of intestinal dysbiotic changes is the use of probiotic drugs. Studies show that these drugs are efficient in both non-communicable diseases and diseases caused by infectious agents. There is data on using probiotics in certain parasitic infestations, but insufficient on their effectiveness in mixed invasion in adults [10, 11].

The aim of the study was to investigate the effectiveness of probiotics containing *Saccharomyces bouvardias CNCM I-745* on the intestinal microbiota in the complex therapy of patients with giardiasis, ascariasis, and mixed invasion.

## Material and methods

90 patients with a mean age of 32.43±1.18 years were under medical supervision. 43 (47.8%) were men and 47 (52.2%) women. The diagnosis of giardiasis and ascariasis was confirmed by detecting the pathogen using a parasitoscopic study of the fecal sample [7]. Patients were divided into 3 groups, equivalent in age and sex, depending on the invasion. Group 1 included patients with giardiasis (n=30), patients with ascariasis (n=30) were included in group 2, and group 3 consisted of patients with mixed invasion. Each group was divided into 2 subgroups, depending on the type of treatment. The first (a) subgroup (n=15) included patients who received traditional basic treatment (included enterosorbents, enzymes, spasmolytics and proper diet) and etiotropic treatment (ornidazole (1a), albendazole (2a) or both (3a). The second (b) subgroup (n=15) consisted of patients whose basic therapy included additional probiotic containing *Saccharomyces bouvardias CNCM I-745* (hereinafter – probiotic) directed for intestine microbiota changes. It was administered orally, 1 capsule 2 times a day for 14 days [3]. 1 capsule contains 250 mg of *Saccharomyces bouvardias CNCM I-745* at least 6×10^6^ CFU and excipients. The control group consisted of 20 healthy individuals. We used the pure cultural method of feces to determine the population level of microbiota by V.A. Znamensky [7]. The study was performed twice: before treatment and 14 days after treatment. The statistical analysis of results was performed using the variational-statistic analysis method.

### Statistical analysis

The evaluation of the research results was carried out using descriptive statistics. Data were checked for the type of distribution using the Shapiro-Wilk test; therefore, the arithmetic mean and error were chosen to represent the normal trend (typical values). Accordingly, the reliability of the data difference in the comparison groups was assessed using the Student's parametric t-test. The results were considered reliable at p<0.05.

## Results

According to fecal microbial analysis, some changes in the species composition of the intestinal microbiota in two subgroups of patients with giardiasis were revealed (Table 1).

**Table 1:** Quantitative composition of microbiota in patients with giardiasis.

Groups of microorganisms	Number of microorganisms, lg CFU/g, M±m			
	Before treatment (n=30)	After treatment		Control group (n=20)
		1a group (n=15)	1b group (n=15)	
***Bifidobacterium spp***.	6.89±0.10*	7.63±0.27** ^#^	7.99±0.22***	8.05±0.15
***Lactobacillus spp***.	6.41±0.13*	7.70±0.19** ^#^	7.97±0.23***	8.10±0.18
***Peptostreptococcus spp***.	6.24±0.12	6.01±0.15	5.72±0.18	5.65±0.20
***Bacteroides spp***.	7.61±0.14*	7.13±0.37** ^#^	7.01±0.22***	6.85±0.20
***E. coli* (total)**	6.87±0.10*	6.98±0.17** ^#^	7.43±0.21***	7.50±0.20
**Weak ferm. *E. coli***	0.83±0.09*	0.69±0.13** ^#^	0.51±0.14***	0.45±0.11
**Hemolytic *E. coli***	0.61±0.10*	0.37±0.14** ^#^	0.05±0.10***	0
***Enterococcus spp***.	7.09±0.13*	7.22±0.25** ^#^	7.83±0.23***	7.95±0.14
**Opportunistic *Enterobacteriaceae spp***.	5.17±0.20*	4.21±0.21** ^#^	4.11±0.21***	4.05±0.27
***Candida spp***.	2.26±0.15*	2.05±0.35** ^#^	1.28±0.20***	1.25±0.18
***Staphylococcus spp***.	2.41±0.18	2.44±0.31	2.30±0.20	2.20±0.12

* – p<0.05 the reliability of the difference between the similarity indicators between the group before treatment and the control group; ** – p<0.05 the reliability of the difference between the similar indicators between the group before treatment and the 1a group; *** – p<0.05 the reliability of the difference between the similar indicators between the group before treatment and the 1b group; ^#^ – p<0.05 the reliability of the difference between the similar indicators between the 1a and 1b groups.

In the group of patients with mixed invasion, the following changes in the intestinal microbiota composition before treatment were observed. The number of *Bifidobacterium spp*., *Lactobacillus spp*., *Enterococcus spp*. and the total content of *E. coli* was reduced. At the same time, there was an increase in the content of *Bacteroides spp*., *E. coli* with low enzymatic properties, hemolytic *E. coli*, opportunistic *Enterobacteriaceae spp*. and *Candida spp*. After treatment of patients in group 1a, the content of *Bifidobacterium spp*. was slightly reduced and amounted to 7.63±0.27 lg CFU/g (p<0.05). The quantitative composition of *Lactobacillus spp*. was 7.70±0.19 lg CFU/g, but it was higher than before treatment (p<0.05). Patients had a reduced number of *Bacteroides spp*. (7.13±0.37 lg CFU/g); the total number of *E. coli* was 6.98±0.17 lg CFU/g, although its content was higher than before treatment (0<0.05). An increased level of *E. coli* with low enzymatic properties was noted, which was 0.69±0.13 lg CFU/g (p<0.05). Hemolytic *E. coli* (0.37±0.14 lg CFU/g) was revealed, as well as the number of *Enterococcus spp*. (7.22±0.25 lg CFU/g) (p<0.05). The opportunistic *Enterobacteriaceae spp*. content in patients was 4.21±0.21 lg CFU/g (p<0.05), including *Klebsiella* and *Proteus*. There were *Candida spp*. in the amount of 2.05±0.35 lg CFU/g (p<0.05). In group 1b, patients had a significant improvement in the microbiota. The content of *Bifidobacterium spp*. was 7.99±0.22 lg CFU/g, which was significantly higher compared to the other two groups (p<0.05). The quantitative composition of *Lactobacillus spp*. was slightly reduced and was equal to 7.97±0.23 lg CFU/g (p<0.05). The number of *Bacteroides spp*. was normalized (7.01±0.22 lg CFU/g) (p<0.05). The total content of *E. coli* (7.43±0.21 lg CFU/g) increased; there was a decrease in the content of *E. coli* with low enzymatic properties (0.51±0.14 lg CFU/g) (p<0.05). Besides, the hemolytic *E. coli* was detected only in one patient (0.05±0.12 lg CFU/g) (p<0.05). Almost complete normalization of *Enterococci spp*. levels were observed (7.83±0.23 lg CFU/g (p<0.05). There was a decrease in the opportunistic *Enterobacteriaceae spp*. content (4.11±0.21 lg CFU/g (p<0.05). The level of *Candida spp*. was minimal (1.28±0.20 lg CFU/g), comparing similar results of other groups (p<0.05).

According to fecal microbial analysis, changes in the species composition of the intestinal microbiota were detected in two subgroups of patients with ascariasis (Table 2).

**Table 2:** Quantitative composition of microbiota in patients with ascariasis.

Groups of microorganisms	Number of microorganisms, lg CFU/g, M±m			
	Before treatment (n=30)	After treatment		Control group (n=20)
		2a group (n=15)	2b group (n=15)	
***Bifidobacterium spp***.	6.30±0.10*	7.63±0.27** ^#^	7.98±0.22***	8.05±0.15
***Lactobacillus spp***.	6.07±0.12*	7.70±0.19** ^#^	7.94±0.23***	8.10±0.18
***Peptostreptococcus spp***.	6.77±0.10	6.01±0.15	5.76±0.18	5.65±0.20
***Bacteroides spp***.	8.09±0.17*	7.13±0.37** ^#^	7.02±0.22***	6.85±0.20
***E. coli* (total)**	6.36±0.09*	6.98±0.17** ^#^	7.32±0.21***	7.50±0.20
**Weak ferm. *E. coli***	0.95±0.09*	0.69±0.13** ^#^	0.55±0.14***	0.45±0.11
**Hemolytic *E. coli***	0.86±0.11*	0.37±0.14** ^#^	0.11±0.10***	0
***Enterococcus spp***.	6.98±0.12*	7.22±0.25** ^#^	7.73±0.23***	7.95±0.14
**Opportunistic *Enterobacteriaceae spp***.	5.45±0.11*	4.21±0.21** ^#^	4.09±0.21***	4.05±0.27
***Candida spp***.	2.52±0.14*	2.05±0.35** ^#^	1.30±0.20***	1.25±0.18
***Staphylococcus spp***.	2.70±0.19	2.44±0.31	2.31±0.20	2.20±0.12

* – p<0.05 the reliability of the difference between the similarity indicators between the group before treatment and the control group; ** – p<0.05 the reliability of the difference between the similar indicators between the group before treatment and the 2a group; *** – p<0.05 the reliability of the difference between the similar indicators between the group before treatment and the 2b group; ^#^ – p<0.05 the reliability of the difference between the similar indicators between the 2a and 2b groups.

In the group of patients with ascariasis, changes in the composition of the intestinal microbiota before treatment were observed. The content of *Bifidobacterium spp*., *Lactobacillus spp*., *Enterococcus spp*., and the total content of *E. coli* was reduced. At the same time, there was an increase in the content of *Bacteroide spp*., *E. coli* with low enzymatic properties, hemolytic *E. coli*, opportunistic *Enterobacteriaceae spp*. and *Candida spp*. After treatment of patients in group 2a, the content of *Bifidobacterium spp*. was slightly reduced and amounted to 7.63±0.27 lg CFU/g (p<0.05). The quantitative composition of *Lactobacillus spp*. was 7.70±0.19 lg CFU/g, but it was higher than before treatment (p<0.05). Patients had a reduced number of *Bacteroides spp*. (7.13±0.37 lg CFU/g), the total number of *E. coli* was 6.98±0.17 lg CFU/g, and its content was higher than before treatment (p<0.05). An increased level of *E. coli* with low enzymatic properties was noted (0.69±0.13 lg CFU/g) (p<0.05). Hemolytic *E. coli* (0.37±0.14 lg CFU/g) and *Enterococci spp*. (7.22±0.25 lg CFU/g) were revealed (p<0.05). The opportunistic *Enterobacteriaceae spp*. content in patients was 4.21±0.21 lg CFU/g (p<0.05), including *Klebsiella, Citrobacter*, and *Proteus*. There were *Candida spp*. in the amount of 2.05±0.35 lg CFU/g (p<0.05). In group 2b, patients showed significant signs of improvement in the microbiota. The content of *Bifidobacterium spp*. was 7.98±0.22 lg CFU/g, which was significantly higher compared to the other two groups (p<0.05). The quantitative composition of *Lactobacillus spp*. was slightly reduced and was equal to 7.94±0.23 lg CFU/g (p<0.05). The number of *Bacteroides spp*. was normalized (7.02±0.22 lg CFU/g) (p<0.05). The total content of *E. coli* (7.32±0.21 lg CFU/g) increased, and there was a decrease in the content of *E. coli* with low enzymatic properties (0.55±0.14 lg CFU/g) (p<0.05). Besides, the hemolytic *E. coli* was detected only in one patient (0.11±0.12 lg CFU/g) (p<0.05). Almost complete normalization of *Enterococci spp*. levels were observed (7.73±0.23 lg CFU/g (p<0.05). The opportunistic *Enterobacteriaceae spp*. content decreased (4.09±0.21 lg CFU/g (p<0.05). The level of *Candida spp*. was minimal (1.30±0.20 lg CFU/g), comparing similar results of other groups (p<0.05).

According to the microbiological study of feces, some changes in the species composition of intestinal microbiota in patients with mixed invasion (Table 3) were revealed.

**Table 3:** Quantitative composition of microbiota in patients with mixed invasion.

Groups of microorganisms	Number of microorganisms, lg CFU/g, M±m			
	Before treatment (n=30)	After treatment		Control group (n=20)
		3a group (n=15)	3b group (n=15)	
***Bifidobacterium spp***.	4.61±0.19*	6.93±0.27** ^#^	7.93±0.22***	8.05±0.15
***Lactobacillus spp***.	4.57±0.20*	6.79±0.19** ^#^	7.64±0.23***	8.10±0.18
***Peptostreptococcus spp***.	8.11±0.29*	6.21±0.15	5.86±0.18	5.65±0.20
***Bacteroides spp***.	9.71±0.22*	7.93±0.37** ^#^	7.07±0.22***	6.85±0.20
***E. coli* (total)**	5.11±0.17*	6.57±0.17** ^#^	7.21±0.21***	7.50±0.20
**Weak ferm. *E. coli***	1.32±0.15*	1.07±0.13** ^#^	0.57±0.14***	0.45±0.11
**Hemolytic *E. coli***	2.21±0.25*	0.57±0.14** ^#^	0.14±0.10***	0
***Enterococcus spp***.	5.75±0.20*	6.93±0.25** ^#^	7.64±0.23***	7.95±0.14
**Opportunistic *Enterobacteriaceae spp***.	7.11±0.36*	5.21±0.21** ^#^	4.21±0.21***	4.05±0.27
***Candida spp***.	5.32±0.33*	2.86±0.35** ^#^	1.36±0.20***	1.25±0.18
***Staphylococcus spp***.	3.96±0.20*	2.64±0.31	2.36±0.20	2.20±0.12

* – p<0.05 the reliability of the difference between the similarity indicators between the group before treatment and the control group; ** – p<0.05 the reliability of the difference between the similar indicators between the group before treatment and the 3a group; *** – p<0.05 the reliability of the difference between the similar indicators between the group before treatment and the 3b group; ^#^ – p<0.05 the reliability of the difference between the similar indicators between the 3a and 3b groups.

Patients in this group had changes in the composition of the intestinal microbiota before treatment. The content of *Bifidobacterium spp*., *Lactobacillus spp*., *Enterococcus spp*., and the total content of *E. coli* was reduced. At the same time, there was an increase in the content of *Peptostreptococcus, Bacteroides, E. coli* with low enzymatic properties, hemolytic *E. coli*, opportunistic *Enterobacteriaceae spp*., *Candida spp*. and *Staphylococci spp*. After treatment, positive changes were observed in both study subgroups. Thus, in patients from group 3a the content of *Bifidobacterium spp*. was slightly reduced (6.93±0.27 lg CFU/g) (p<0.05). The quantitative composition of *Lactobacillus spp*. was 6.79±0.19 lg CFU/g, but it was higher than before treatment (p<0.05). There was also a small amount of *Peptostreptococcus spp*. (6.21±0.15 lg CFU/g) and *Bacteroides spp*. (7.93±0.37 lg CFU/g) (p<0.05). Patients had a reduced total number of *E. coli* (6.57±0.17 lg CFU/g), although its content was higher than before treatment (p<0.05). An increased level of *E. coli* with low enzymatic properties was noted, which was 1.07±0.13 lg CFU/g (p<0.05). There was hemolytic *E. coli* (0.57±0.14 lg CFU/g), which is not normally registered (p<0.05), as well as the number of *Enterococci spp*. (6.93±0.25 lg CFU/g) (p<0.05). The opportunistic *Enterobacteriaceae spp*. content after treatment was 5.21±0.21 lg CFU/g (p<0.05) and included *Klebsiella, Enterobacter, Serratia, Citrobacter*, and *Proteus*. There were *Candida spp*. in the amount of 2.86±0.35 lg CFU/g (p<0.05). There was a decrease in the number of *Staphylococci spp*. (2.64±0.31 lg CFU/g), compared with the group before treatment (p<0.05). In group 3b (treatment with probiotics addition), there were more significant signs of improvement in the intestinal microbiota concerning the results obtained before treatment and compared with patients' data in group 3b. The content of *Bifidobacterium spp*. was 7.93±0.22 lg CFU/g, which was significantly higher compared to the other two groups. The quantitative composition of *Lactobacillus spp*. was slightly reduced –7.64±0.23 lg CFU/g (p<0.05). Among other representatives of the anaerobic intestinal flora, the number of *Bacteroides* changed positively (7.07±0.22 lg CFU/g) (p<0.05), and the number of *Peptostreptococcus spp*. (5.86±0.18 lg CFU/g) was insignificantly lower than in patients from group 3a (p>0.05). The number of *E. coli* also changed in this group of patients: the total content of *E. coli* (7.21±0.21 lg CFU/g) increased, compared with data before treatment and without probiotics use (p<0.05). There was a decrease in the content of *E. coli* with low enzymatic properties (0.57±0.14 lg CFU/g) (p<0.05). In addition, hemolytic *E. coli* was detected only in 2 patients (0.14±0.12 lg CFU/g), which is significantly lower than that before treatment (p<0.05) and compared with patients in group 3a (p<0.05).

Analyzing the content of other representatives of the anaerobic spectrum of the intestinal microbiota, almost complete normalization of the level of *Enterococcus spp*. was observed (7.64±0.23 lg CFU/g) (p<0.05). Against the background of treatment with probiotics, a decrease in the content of opportunistic *Enterobacteriaceae spp*. was noticed, equal to 4.21±0.21 lg CFU/g (p<0.05). The level of *Candida spp*. was minimal (1.36±0.20 lg CFU/g), comparing similar results of other groups (p<0.05). There was also a decrease in the content of *Staphylococci spp*. (2.36±0.20 lg CFU/g), however, the results were not reliable for the group without probiotics (p>0.05).

## Discussion

Following the analysis of system indicators of the intestinal microflora, we observed an increase in the content of autochthonous intestinal flora (lactobacteria, bifidobacteria, the total number of *Escherichia* and *enterococci*), and a decrease in the number of allochthonous microorganisms (*Bacteroides, Escherichia* with weakly fermentative properties, UPE and fungi of the genus *Candida*) and hemolytic *Escherichia coli* [12–14].

The use of probiotics containing Saccharomyces's bouvardia has proven effective in various conditions accompanied by intestinal dysbiosis, and in combination with the mediated effect of silymarin, its level increases [15, 16].

The most significant changes related to the state of intestinal microflora were the increase in the content of autochthonous intestinal flora (lactobacteria, bifidobacteria, the total number of *Escherichia* and *enterococci*), and a decrease in the number of allochthonous microorganisms (*Bacteroides, Escherichia* with weakly fermentative properties, UPE and fungi of the genus *Candida*) and hemolytic *Escherichia coli* [17–19]. The use of probiotics containing Saccharomyces's bouvardia has proven effective in various conditions accompanied by intestinal dysbiosis [6, 20, 21].

## Conclusions

Dysbiotic changes in the intestinal microbiota were observed in patients with giardiasis and ascariasis and were most pronounced in their mixed invasion, which was characterized by a decrease in the level of normal intestinal microbiota (*Bifidobacterium spp*. – 4.61±0.19 lg CFU/g, *Lactobacillus spp*. – 4.57±0.20 lg CFU/g), an increased content of opportunistic *enterobacteria* included *Klebsiella, Enterobacter, Serratia, Citrobacter, Proteus* (7.11±0.36 lg CFU/g), fungal flora (5.32±0.33 lg CFU/g) and *Staphylococci spp*. (3.96±0.20 lg CFU/g). Basic treatment of patients with giardiasis, ascariasis and mixed invasion was accompanied by improvement in some indicators of the intestinal microbiota, compared with the data before treatment (*Bifidobacterium spp*. – 6.93±0.27 lg CFU/g) (p<0.05), *Lactobacillus spp*. – 6.79±0.19 lg CFU/g (p<0.05), *Candida spp*. – 2.86±0.35 (p<0.05).

The use of probiotics containing Saccharomyces bouvardia CNCM I-745 in the complex therapy of patients with mixed invasion of giardiasis and ascariasis increased treatment efficiency given the significant improvement in the intestinal microbiota (*Bifidobacterium spp*. – 7.93±0.22 lg CFU/g) (p<0.05), *Lactobacillus spp*. – 7.64±0.23 lg CFU/g (p<0.05), *Candida spp*. – 1.36±0.20 (p<0.05), *Staphylococci spp*. – 2.36±0.20 lg CFU/g (p<0.05) compared to treatment results and with a similar group without use.

## Data Availability

The data of this study is available by request.
